# ProbFAST: Probabilistic Functional Analysis System Tool

**DOI:** 10.1186/1471-2105-11-161

**Published:** 2010-03-30

**Authors:** Israel T Silva, Ricardo ZN Vêncio, Thiago YK Oliveira, Greice A Molfetta, Wilson A Silva

**Affiliations:** 1 Department of Genetics, Faculty of Medicine, University of São Paulo, Ribeirão Preto, Brazil; 2 National Institute of Science and Technology in Stem Cell and Cell Therapy, Center for Cell Therapy and Regional Blood Center, Ribeirão Preto, Brazil

## Abstract

**Background:**

The post-genomic era has brought new challenges regarding the understanding of the organization and function of the human genome. Many of these challenges are centered on the meaning of differential gene regulation under distinct biological conditions and can be performed by analyzing the Multiple Differential Expression (MDE) of genes associated with normal and abnormal biological processes. Currently MDE analyses are limited to usual methods of differential expression initially designed for paired analysis.

**Results:**

We proposed a web platform named ProbFAST for MDE analysis which uses Bayesian inference to identify key genes that are intuitively prioritized by means of probabilities. A simulated study revealed that our method gives a better performance when compared to other approaches and when applied to public expression data, we demonstrated its flexibility to obtain relevant genes biologically associated with normal and abnormal biological processes.

**Conclusions:**

ProbFAST is a free accessible web-based application that enables MDE analysis on a global scale. It offers an efficient methodological approach for MDE analysis of a set of genes that are turned on and off related to functional information during the evolution of a tumor or tissue differentiation. ProbFAST server can be accessed at http://gdm.fmrp.usp.br/probfast.

## Background

Transcriptome analysis of a tissue or cell type has been widely used since the development of methodological approaches for the large-scale study of gene expression such as SAGE [1], MPSS [2], Microarray [3]. The next-generation sequencing technology has been adapted to transcriptome analysis and the ability to accurately measure mRNA signals must provide unprecedented impact on gene expression analysis [4,5]. Thus, it is accepted that high-throughput data represents the starting point to predict further our understanding of molecular disorders associated with the physiopathology of a given phenotype.

The most classical application to the analysis of gene expression focuses on the identification of genes differentially expressed between two biological conditions. At this stage, a large number of statistical tests is used for a precise identification of candidate genes [6,7]. The network of biological processes involved in the evolution of a tumor or in tissue differentiation is extremely complex and requires the development of mathematical models for a simultaneous analysis of a set of genes in two or more biological conditions. Analyses of this nature are currently performed using standard methods designed for paired analyses. Thus, it is highly necessary to develop methods for analysis of multiple expression of a gene. We shall define the approach in the current study as Multiple Differential Expression (MDE).

An example of the application of MDE approach may be illustrated by the following question: what genes have shown an increasing level of expression in three libraries (A, B and C) representing the stages (evolution) of a tumor? To answer this question, the usual procedure analyses couples of libraries separately and makes conjunctions or disjunctions of the relations found, e.g. A > B AND B > C. In fact, this analysis is traditionally used to select any *g *gene with an expression profiles such as *A*_*g *_>*B*_*g *_>*C*_*g*_. In this type of paired analysis, the main problem is the sensitivity and specificity of statistical tests used to detect what genes are differentially expressed [8]. These statistical measures are closely related to the concepts of type I and type II errors and they are potentiated when more than two biological conditions are analyzed simultaneously. To address this shortfall, we introduced a Bayesian model to compute the generalization of the pairwise comparisons in order to perform MDE analysis. It is a new probabilistic method for targeted gene selection on two or more classes through an intuitive approach involving a question formulation process, and a probability linked to it. In summary, all genes in accordance with the previously formulated question will be ordered on the basis of the probability that the question is true.

We presented a web-based system named Probabilistic Functional Analysis System Tool (ProbFAST) that permits suitable MDE analysis on a global scale. This tool differs from others [8-11] by permitting the investigator to analyze the global gene expression in different biological conditions using private and/or public data, integrating it into a set of functional pieces of information including Gene Ontology [12], KEGG [13] and Biocarta [14]. Within this context, the tool becomes useful for the disclosure of genes related to biological processes that are active during the cell differentiation and growth, as well as during organogenesis. ProbFAST is designed primarily for sequencing-based data, including data from next-generation sequencing technology.

## Implementation

### Design functionality

ProbFAST is a tool which uses the client-server architecture [Additional file 1: Supplemental Figure 1]. The back-end consists of a set of MySQL [15] relational tables that store functional information extracted from the KEGG, BioCarta and Gene Ontology repositories. Furthermore, all the expression data of Gene Expression Omnibus (GEO) [16] generated by the counting technique are stored, including 1,800 SAGE and MPSS libraries of approximately 40 species. All databases are monthly updated, ensuring the access to the most recent information. The server side is composed of three main interfaces that enable remote use with convenient data uploading and result visualization features.

**Figure 1:**
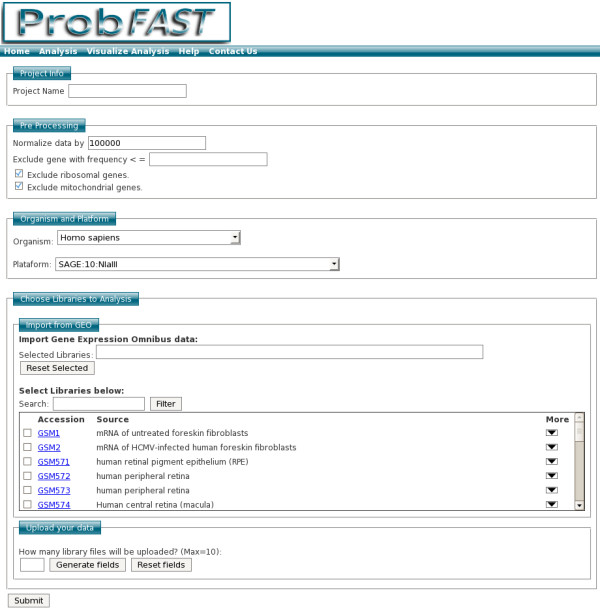
**Screenshot of ProbFAST input data page**. To add a new analysis, the user must add the project name and set up the pre processing information (optional). Next, the user must choose organism, platform and loading gene expression sample from GEO and/or upload the data.

The analysis starts with a friendly interface for the inclusion of the project name and parameters to the preprocessing and upload of libraries (Figure 1). In the upload process, two options are available: 1) import data from GEO: a search interface allows displaying a list of expression profile experiments related to organism and keywords filter, and 2) the upload option to analyze a new experiment that is not included in the GEO database. To do that, the user needs to submit a file with a predefined format (detailed information on file format is available at the help page). The file may be uploaded compressed in gz, zip, or rar format. The gene identifiers supported by ProbFAST include NCBI ID, gene symbol, tag sequence or Unigene accession.

**Figure 2:**
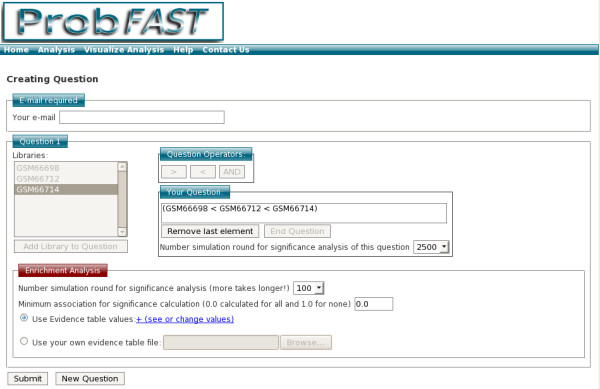
**Creating question**. Now, the user needs to specify one or more target question. For that, work layer allows choosing sample and question operators to help creating the question. Double click on the sample name allows set pool analysis.

**Figure 3:**
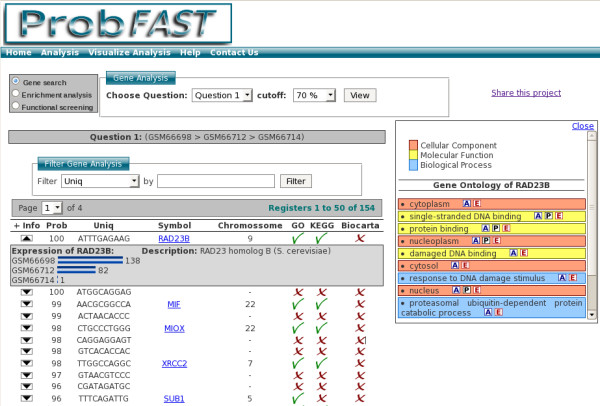
**Gene search**. Firstly, users can select the question target and cutoff value through the *Gene Search *panel. Then the genes associated question and cutoff will be showed. By clicking on any image from functional categories it enables the user to visualize indexed information on the layer in the right side. Key-word filters can be applied in order to refine results.

**Figure 4:**
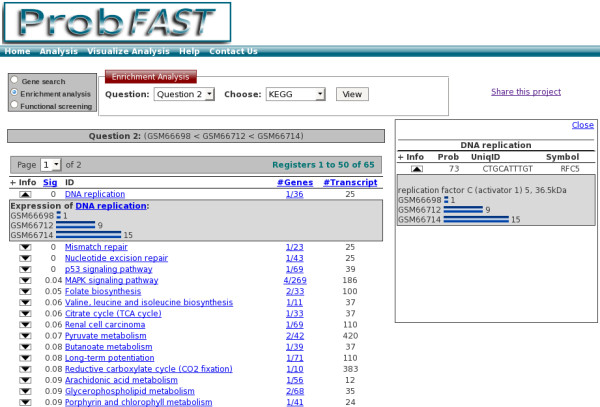
**Enrichment analysis**. An example of the enriched functional term list output for KEGG category. The resulting enriched KEGG pathways can be visualized using a table consisting of enrichment cutoff values of the enriched terms (with web links to additional information), total of the relevant annotated genes and counting of transcripts.

**Figure 5:**
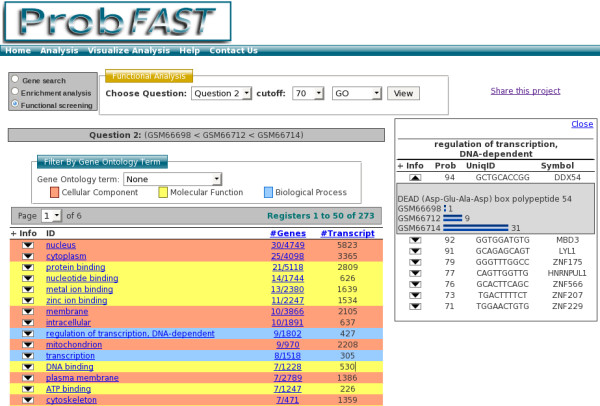
**Functional Screening**. The functional categories presenting the largest number of genes with values above the cutoff related to the question will be listed here.

After the submission, users must formulate question(s) by a comprehensive frame box and define the parameters for enrichment analysis (Figure 2). The parameters are preconfigured and can be adjusted according to their stringency criterion. Finally, after processing the user will be informed about the result by e-mail. The results are provided in three analysis aspects:

• Gene search: The user can select one of the questions formulated and adjust the probability (cutoff) of interest. All genes will be listed according to the cutoff related to the question (Figure 3).

• Enrichment analysis: This option permits the user to select the question formulated and the functional category of interest. The result will reveal the enriched functional categories ordered according to the level of significance (Sig) (Figure 4).

• Functional screening: The functional categories with the largest number of genes with probability above the predefined cutoff will be listed here (Figure 5).

In the options **Enrichment analysis **and **Functional screen**, the functional categories can be ordered according to the total expression level, total number of genes and enrichment score (only **Enrichment analysis**).

By clicking on the +*Info *key, a window will appear with the sum of the expression levels of the genes related to the formulated question. Several links will permit the user to access external information about the genes and the functional categories.

The web interface is implemented in the PERL language and the Common Gateway Interface (CGI) protocol was used to permit the access to the services of data submission and result visualization. The web interface is based on the new technology Web 2.0 AJAX.

### Statistical analysis

The data obtained by counting techniques such as SAGE, MPSS, RNAseq and the next-generation sequencing technologies generate a simple enumeration measurement that can be modeled in a probabilistic manner. The expression of a given gene *G *(which, for the sake of simplicity will be considered implicitly pre-determined and the extra notation avoided) in the *i*^*th *^experiment is estimated by the abundance of messenger RNA levels (mRNAs) *π *and is commonly modeled by a Bernoulli Process [17]. This means that the probability of observing a tag for that gene is proportional to *π* and we see the sequencing processes as if it was a urn. Inside this imaginary urn, the different colored balls would represent different tags and the number of each ball in the urn is proportional to the mRNA abundance. If we knew *π*, it would be easier to determine the probability *L *of observing *N *tags after sequencing a total of *T *tags:(1)

where *N *are the counts for a specific tag that is the proxi for the considered gene, *T *is the total number of sequenced tags and *C *is a constant for which the actual value is not relevant.

The following urn-like model is well-known in Bayesian statistics and the inference about the abundance *π *is performed, with the aid of an uniform prior, using Baye's rule:(2)

which can be re-written as:(3)

where *a *= *N *+ 1, *b *= *T *- *N *+ 1 and *B*(*a*, *b*) = .

or in usual statistical notation:(4)

Here we extended this basic model by considering a mixture of beta densities as a way to encode the information from several sequenced libraries (e.g. patients, individuals, treatment, etc) from the same group (e.g. cancer, normal, drugX, etc):(5)

where(6)

*B *is the beta special function, *a*_*i *_= *N*_*i *_+ 1, *b*_*i *_= *T*_*i *_- *N*_*i *_+ 1, the sub-index *i *denotes the *i*^*th *^library and *m *is the number of libraries that compose a single group, i.e. the components of the mixture. Note that there is no sub-index to denote a gene (or tag) in these equations because it is implicit that a given gene is our focus.

It is somewhat easy to derive an estimate for a given probabilistic question provided that one knows how to generate random deviates from all atomic parts of the question. If the group at hand is composed of just one library *m *= 1, then the model reduces itself to the usual beta random variable which can be easily simulated. On the other hand, for a real mixture (e.g. more than 1 patient in the class "cancer", for example) *m *> 1, the simulation can be more computationally expensive, but also easy. Our mathematical code implemented in R language automatically switches between these two modes and simulates random variables properly.

Once a sufficient amount of random variables for all atomic elements in the probabilistic question are in place, the probability estimation is trivial: , where *S*_*t *_is simulations number for which the question returns **true **and *S*_*n *_is the total number of simulations.

To assess the efficiency of our method, comparing it to approaches reported in the literature [8,18-20], we performed a simulation study [Additional file 1: Supplemental Figure 2 and Figure 3]. The sensitivity and specificity of all methods were evaluated and compared by receiver operating characteristic (ROC) curves [21].

To perform MDE analysis with data generated by counting techniques, we developed the probMDE algorithm to compute the generalization of the paired analyses according to the probabilistic model above. The R code, with the complete and self-contained implementation of our model, can be downloaded in [22].

Next, using these probabilistic results, we incorporated enrichment analysis for the functional categories Gene Ontology, KEGG, and BIOCARTA using the methods and source-code available by the tool ProbCD [23]. This is an efficient approach for considering the rate of error included in all annotation process [24]. The user can also upload other probabilistic annotation values for the functional categories available in ProbFAST. The statistical significance for the enrichment is obtained as in ProbCD tool [23]: a null distribution for the statistical association measure is created using a randomization approach. The measured association is then compared with the null to derive p-values. A term is significantly over-represented (or equivalently, the gene list is enriched) depending on the user-defined thresholds for significance and/or association.

## Results and Discussion

To evaluate the efficacy of ProbFAST in order to provide meaningful insights biologically, we first compared the results obtained by ProbFAST with data previously reported in the literature [25]. We also found new targets when reanalyzing public dataset. We showed three of several applications of ProbFAST. All the results presented can be accessed by PID 291347212008, 231240412009 and 11850212008 respectively at the *Visualize Analysis *section at the website of the project [26].

### 1) Up and down-regulation

Lee et al [25] measured the effect of radiofrequency (RF) on gene expression at the genome level. They observed that using RF of 2.45 GHz, 221 genes presented abnormal gene expression after 2h of exposure and 759 genes showed abnormal expression after 6h of exposure. Using gene expression as the indicator to determine if there were any biological effect of RF, three samples were used for the analysis: a control with 2h sham exposure (GSM66698), a sample of HL-60 cells exposed for 2h (GSM66712) and a sample exposed for 6h (GSM66714).

We used the same data set and raised two questions to offer a genome-wide scenario of genes that were up-regulated on (*GSM*66698 <*GSM*66712 <*GSM*66714) and down-regulated on (*GSM*66698 >*GSM*66712 >*GSM*66714). We launched into ProbFAST all the tags reported by those authors and we divided the results into up and down-regulated ones. Afterwards, we selected the first 50 genes with a cutoff > = 70 criterion from each group obtained by our analysis and compared them to the genes reported by the authors. We were able to obtain the same genes reported by Lee et al [25] and, fortunately, we were able to observe new genes that have not been described by those authors. Such genes changed their expression due to RF exposure. Among the new genes we would like to describe PTMA and EIF5 genes that were down and up-regulated respectively.

Prothymosin-alpha or PTMA (MIM: 188390) was identified in the thymus gland while producing several hormones or hormone-like substances known as prothymosin-alpha. Regarding its role, Jiang et al [27] identified a pathway that regulates mitochondria-initiated caspase activity. In this pathway, PTMA negatively regulates caspase-9 activation by inhibiting apoptosome formation. Elimination of PTMA expression by RNA interference sensitized cells to ultraviolet irradiation-induced apoptosis. Another example of the relation between the PTMA gene and the radiosensitivity is the finding of Ojima et al [28], who used microarray to determine if the expression levels of specific genes could predict clinical radiosensitivity in human colorectal cancer. They found that PTMA was up-regulated in the resistant cell lines and suggested that PTMA may be a novel marker for predicting the effectiveness of radiotherapy in clinical cases of colorectal cancer.

The other gene revealed by ProbFAST was eukaryotic translation initiation factor 5 A or EIF5A (MIM:600187). EIF5A is an essential protein tightly linked to cellular homeostasis, and its protein may be also involved in nucleocytoplasmic mRNA transport. Recent studies have indicated that EIF5A may play a role in cell death, as its over-expression was found to induce apoptosis in lung cancer [29]. All these reports corroborate the role of these genes in regulating the apoptosis process. In addition, both are modulated by radiation or RF exposure. These findings are good examples of which genes should be found by a functional analysis tool and we would like to emphasize that ProbFAST was able to detect these genes.

### 2) Next-Generation Sequencing

Currently, the Next-Generation Sequencing is more frequently found in laboratories and these laboratories must be able to deal with a great amount of data from this new high-throughput platform. We carried out an analysis to detect which genes were up-regulated (*N *<*S*2 <*S*4) in human Solexa LongSAGE from Cancer Genome Anatomy Project SAGE library collection. Three samples were used: a control N - skin normal/GSM384135, S2 - stage 2 melanoma skin/GSM384132 and S4 - stage 4 melanoma skin/GSM384133.

We have detected some genes from MAGE family such as MAGED1, MAGED2, MAGEF1, MAGEH1; these genes show that ProbFAST is able to detect genes accurately once they are melanoma associated antigens and they are expected to be found in the question *N *<*S*2 <*S*4. Another expected gene was MCAM which is a melanoma cell adhesion molecule and it was also detected by ProbFAST. Interesting was the detection of RGS1 and SPP1 genes. Both genes were detected as over-expressed genes in melanoma and data from literature has already reported that both genes are considered great markers for melanoma while investigating malignant melanoma and benign nevi [30].

### 3) Analysis of one or more groups of biological replicates

ProbFAST tool is also able to analyze groups of libraries. In order to prove this skill, we selected four SAGE libraries and the analysis was performed while clustering the tumoral colon libraries (GSM383859 and GSM38386) in one group and two normal libraries (GSM383869 and GSM383870) within the other group. The question loaded to ProbFAST was (*GSM*383859, *GSM*383860 >*GSM*383869, *GSM*383870). The analysis was performed with 80% probability cutoff.

We found a group of transcriptions factors and the most expressed were TGFBI, SRCAP, GTF2A1L and SOX12. TGFBI proteins can modulate cell adhesion due to its inhibition [31]. GTF2A1L protein has a role in the assembly and stability of the RNA polymerase II transcription pre-initiation complex on a eukaryotic core promoter [32]. SOX proteins are implicated in cell fate decisions in a wide range of developmental processes. SOX transcription factors have diverse tissue-specific expression patterns during early development and it was proposed that they acted target-specific transcription factors and/or as chromatin structure regulatory elements. SOX12 expression in various tissues also suggests a role in both differentiation and maintenance of several cell types [33].

We also detected a group of genes highly expressed in the tumoral libraries which has a direct role in tumorigenesis. These genes are BRMS1, GREB1 and PRR5. BRMS1 reduces the metastatic potential, but not the tumorogenicity of human breast cancer and melanoma cell lines [34,35]. GREB1 is an estrogen-responsive gene that is an early response gene in the estrogen receptor-regulated pathway. This gene may play an important role in hormone-responsive tissues and cancer [36]. PRR5 gene encodes a protein with a proline rich domain. This gene is located in a region of chromosome 22 reported as containing a tumor suppressor gene that may be involved in breast and colorectal tumorigenesis [37].

Our tool also detected some other genes more expressed in the tumoral libraries and have important roles in cell progression, proliferation and differentiation. These genes are TRIM28, BP1, CAD and S100A6. TRIM28 gene encodes a protein that mediates transcriptional control by interaction with a repression domain found in many transcription factors. There are new findings suggesting that this transcriptional repressor plays a role in cell proliferation [38]. BP1 gene is also a member of a family of translation repressor proteins. This gene has been already reported as having a role in the progression of breast neoplasms through cell signaling [39]. On the other hand, CAD gene encodes a protein which is required by mammalian cells to proliferate [40]. S100A6 proteins are located in the cytoplasm and/or nucleus of a wide range of cells, and are involved in the regulation of a number of cellular processes such as cell cycle progression and differentiation. S100A6 is also associated with human colorectal adenocarcinoma tumorigenesis and invasion/metastasis [41].

## Conclusions

ProbFAST is a web application that facilitates the analysis of enumeration gene expression data generated by high-throughput technologies. It supports a flexible Bayesian method that assigns a probability for each gene analyzed according to a previously defined question, and aims to bring a top-down approach to carry out MDE analysis according to the investigator's background.

## Future directions

ProbFAST currently supports statistical analysis for enumeration data and our goal is to extend this capability for microarray data.

## Availability and requirements

• Project Name: ProbFAST - Probabilistic Functional Analysis System Tool

• Project Home Page: http://gdm.fmrp.usp.br/probfast

• Operating Systems: UNIX-like Platforms

• Programming Languages: Perl and R

• Other requirements: MySQL

• License: GNU General Public License

## Authors' contributions

ITS designed and implemented the project. RZNV proposed the beta-mixture extension and wrote R code. TYKO and ITS set up the web-page interface. GAM and ITS performed and analyzed the data shown in the result section from real data. WAS contributed with ideas and requirements. All authors read and approved the final manuscript.

## Supplementary Material

Additional file 1 **ProbFAST architecture and simulation study**. The file shows the client-server architecture of the tool ProbFAST and ROC curves analysis from simulation study. Click here for file
