# Supplementary material for: ProbFAST: Probabilistic Functional Analysis System Tool
Source: BMC Bioinformatics. 2010 Mar 30;11:161. doi: 10.1186/1471-2105-11-161 (PMC2868004; doi:10.1186/1471-2105-11-161)
Supplement: Additional file 1 — ProbFAST architecture and simulation study. The file shows the client-server architecture of the tool ProbFAST and ROC curves analysis from simulation study. [file 1471-2105-11-161-S1.PDF]

Supplemental Material

ProbFAST Architecture

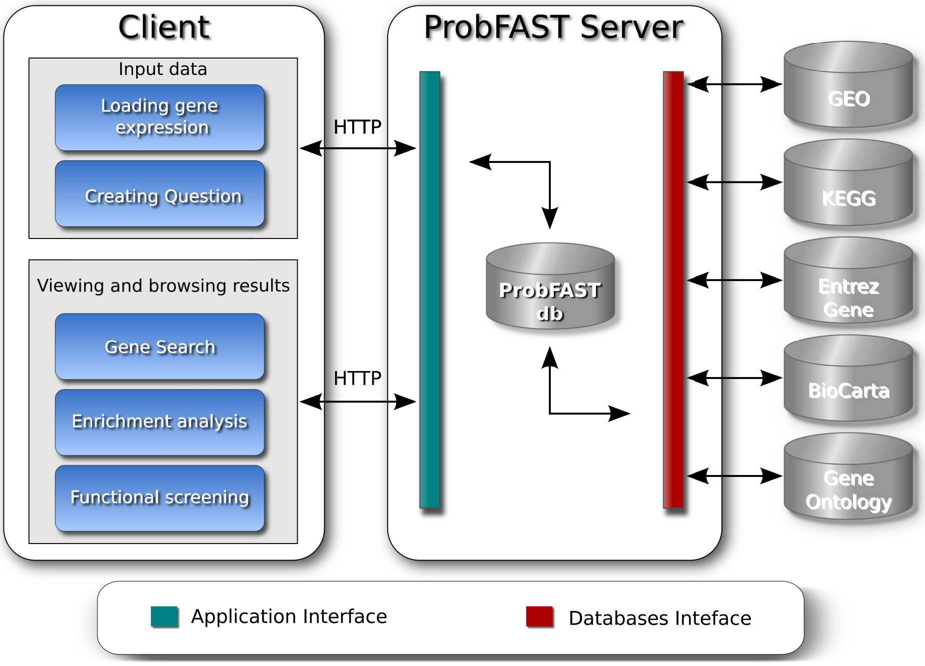

Figure 1

The ProbFAST architecture. The Server side provides *Application interface* through HTTP that enables remote use with convenient data uploading and result visualization features. The *Database Interface* ensuring the access to the most recent functional information and gene expression from BioCarta, KEGG, Gene Ontology, Entrez Gene and Gene Expression Omnibus (GEO).

Simulation study

To compare the efficiency of the method implemented in ProbFAST to perform EDM analysis with other approaches, simulated data were generated and the performance of each method was evaluated. At first our purpose was to identify what g genes presented the gene expression pattern in the samples A, B, C and D such as $A > C > D > B$. Then paired analysis was performed with 2 pools (A and B) with 4 biological samples in each class to indentify what genes presented down-regulation such as $A_1, A_2, A_3, A_4 > B_1, B_2, B_3, B_4$.

In order to perform the simulated analysis, we generated virtual data that represented the transcription level of a gene from the experimental information available in *MAQC* project, an initiative of the American Institution FDA, which gathered 137 participants from 51 organizations [1]. In this project, the gene expression of four samples was measured in six different platforms of Microarray (GSE5350): *Applied Biosystems (ABI)*, *Affymetrix (AFX)*, *Agilent Technologies (AGL for two colors and AG1 for one-color)*, *GE Healthcare (GEH)*, *Illumina (ILM)* and *Eppendorf (EPP)*. The main purpose was to verify if the differential gene expression could be produced among and inside each platform. The specific objectives included providing tools for quality control, presenting guidance for Microarray data analysis and evaluating the performance achieved by various platforms, as well as the advantages and disadvantages of different methods of data analysis.

To generate virtual data from the measure intensity, the following pre processing steps were performed:

1. Build a true table with the names of the differentially expressed genes (TAQ – TaQMain Assays) provided by MAQC project;
2. Recover the data of the first test site from the Affymetrix HG-U133 (GPL570) platform;
3. Attach the most recent note from the Affymetrix *site*[1];
4. Choose the highest intensity probe, for those annotated to the same gene;
5. Exclude all repeated probes.

Through the resulting data of the pre processing steps described above, the abundance of the virtual expression level was defined according to the intensity measure of each spot, *i.e.* considering the following operation [2]:

$$v_{g,i} = \left(\frac{x_{g,i}}{\sum_{g=1}^{n} x_{g,i}}\right) * F \quad (1)$$

where, $x_{g,i}$ is the amount of the measure of intensity of the gene g in sample i, F is the magnitudes number[2] and $v_{g,i}$ is the virtual total number regarding the virtually sequenced tags of the gene g in the sample i.

Four samples were used:

- A, 100% UHRR – *Universal human Reference RNA*;
- B, 100% UBRR – *Human Brain Reference RNA*;
- C, 75% UHRR:25% HBRR;
- D, 25% UHRR:75% HBRR.

The *priori* information about genes differentially expressed (*TAQ – TaQMain assays*) among the samples is crucial for generating the virtual data and ensuring the accuracy of the analyses results. Here the approach becomes more useful because it is corroborated by the previous knowledge using evidential experiments.

ROC curve

The performance of each method was evaluated through receiver operating characteristic (ROC) curves [3]. The ROC curve represents the result of the relation between sensitivity (True Positive Fraction – TPF) and specificity (False Positive Fraction) from the classification tests according to different *cutoff*[3] for each method.

Besides the information of the validated genes – *true table*, six variables were used to build the ROC curve: the significance value (*p-value*) of each method – *threshold*, the *cutoff* value and the four values of the classification test (TP – True Positive, TN – True Negative, FP – False Positive and FN – False Negative). The calculation of the classification test (TP, TN, FP and FN) can be followed according to the pseudo code described in the algorithm 1, see in the Table 1 for the variables used:

[1] http://www.affymetrix.com/Auth/analysis/downloads/na26/ivt/HG-U133_Plus_2.na26.annot.csv.zip

[2] A constant that represents the total of transcripts expected in a library

[3] The cutoff values are numeric spaced intervals that start a *cutoff* acceptable to the most reliable (for example, for the *p-value* all the intervals between 0.05 to 0.001)

```
    input  : C,T,R
    output: cutoff value list and TP, TN, FP and FN values
1   i ← 0;
2   for c ← 1 to length(C) do
3       for t ← 1 to length(T) do
4           if T[t] >= C[c] then
5               if (existsTV(G[t], t) = 1) then
6                   // TP;
7                   R[c][0] ← R[c][0] + 1;
8               else
9                   // FP;
10                  R[c][2] ← R[c][2] + 1;
11          else
12              if (existsTV(G[t], t) = 1) then
13                  // FN;
14                  R[c][3] ← R[c][3] + 1;
15              else
16                  // TN;
17                  R[c][1] ← R[c][1] + 1;
18      // specificity: 1 - TN/(FP + TN);
19      R[c][4] ← 1 − R[c][1]/(R[c][2] + R[c][1]);
20      // sensitivity: TP / (TP + FN);
21      R[c][5] ← R[c][0]/((R[c][0] + R[c][3]);
```

Algorithm 1: Pseudo code of algorithm to compute the TP, TN, FP and FN values

The algorithm evaluates all generated cutoffs (**line 2**). Each value is compared to all the significance values – *threshold* (**lines 4-16**). Next, the values for the classes TP, TF, FP and FN are computed. At the end, each point that corresponds to the relation between specificity (axis x) and the sensibility (axis y) are calculated and stored to build the ROC curve. The function *existsTv* (**lines 5 and 12**) verifies if there is a gene correspondent to the threshold analyzed in the true table.

Analysis of the ROC curves revealed a better prediction (Figure 2) of our method for Multiple Differential Expression (MDE) analysis compared to usual paired analysis. Also, the performance of our method was better for the analysis of biological groups (Figure 3). These results suggest that our method is more robust. Our flexible method is suitable for exploring the gene expression data in the several ways according to a defined question. The analysis of biological groups can be more computationaly expensive, but we are working in the parallel version that can effectively reduce the processing time result.

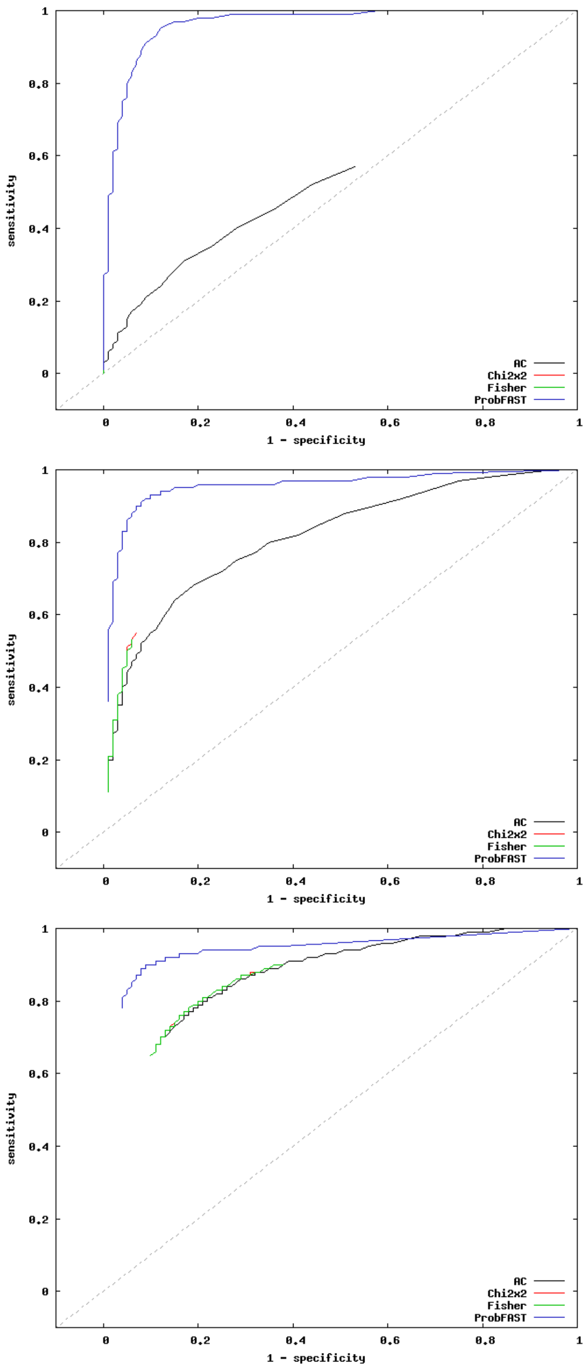

∅ = 1E6

∅ = 1E7

∅ = 1E8

Figure 2

The ROC curves of the four tests based on simulation data to MDE analysis ($A > C > D > B$) with various magnitudes (∅) shown on the right of each graph.

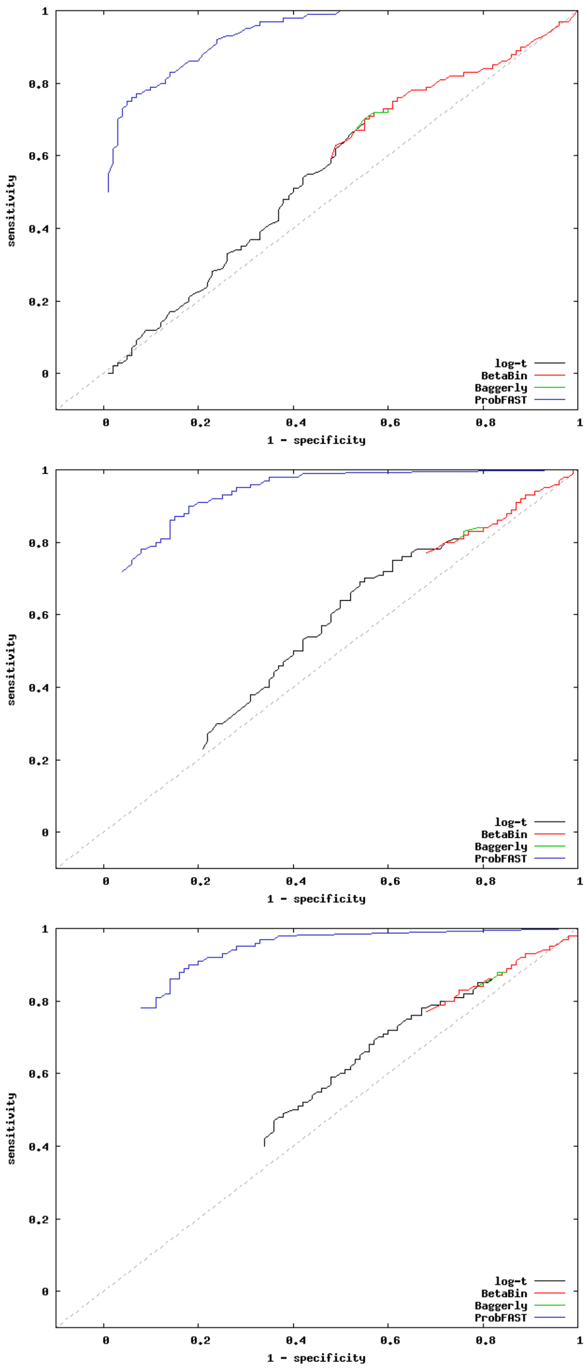

∅ = 1E6

∅ = 1E7

∅ = 1E8

Figure 3

The ROC curves of the four tests based on simulation data to biological groups analysis ($A_1, A_2, A_3, A_4 > B_1, B_2, B_3, B_4$) with various magnitudes (∅) shown on the right of each graph.

Tables

Table 1 - Notations in algorithm to compute TP, TN, FP and FN values

Symbol	Description
C	List of *cutoff* value s
T	List of the all *threshold*
R	Matrix initialized to store the *cutoff* for each classification

References

1. Shi L, Reid LH, Jones WD, Shippy R, Warrington JA, Baker SC, Collins PJ, de Longueville F, Kawasaki ES, Lee KY, Luo Y, Sun YA, Willey JC, Setterquist RA, Fischer GM, Tong W, Dragan YP, Dix DJ, Frueh FW, Goodsaid FM, Herman D, Jensen RV, Johnson CD, Lobenhofer EK, Puri RK, Schrf U, Thierry-Mieg J, Wang C, Wilson M, Wolber PK, Zhang L, Amur S, Bao W, Barbacioru CC, Lucas AB, Bertholet V, Boysen C, Bromley B, Brown D, Brunner A, Canales R, Cao XM, Cebula TA, Chen JJ, Cheng J, Chu TM, Chudin E, Corson J, Corton JC, Croner LJ, Davies C, Davison TS, Delenstarr G, Deng X, Dorris D, Eklund AC, Fan XH, Fang H, Fulmer-Smentek S, Fuscoe JC, Gallagher K, Ge W, Guo L, Guo X, Hager J, Haje PK, Han J, Han T, Harbottle HC, Harris SC, Hatchwell E, Hauser CA, Hester S, Hong H, Hurban P, Jackson SA, Ji H, Knight CR, Kuo WP, LeClerc JE, Levy S, Li QZ, Liu C, Liu Y, Lombardi MJ, Ma Y, Magnuson SR, Maqsodi B, McDaniel T, Mei N, Myklebost O, Ning B, Novoradovskaya N, Orr MS, Osborn TW, Papallo A, Patterson TA, Perkins RG, Peters EH, Peterson R, Philips KL, Pine PS, Pusztai L, Qian F, Ren H, Rosen M, Rosenzweig BA, Samaha RR, Schena M, Schroth GP, Shchegrova S, Smith DD, Staedtler F, Su Z, Sun H, Szallasi Z, Tezak Z, Thierry-Mieg D, Thompson KL, Tikhonova I, Turpaz Y, Vallanat B, Van C, Walker SJ, Wang SJ, Wang Y, Wolfinger R, Wong A, Wu J, Xiao C, Xie Q, Xu J, Yang W, Zhang L, Zhong S, Zong Y, Slikker W: **The MicroArray Quality Control (MAQC) project shows inter- and intraplatform reproducibility of gene expression measurements**. *Nat. Biotechnol.* 2006, **24**:1151–1161.

2. Vêncio RZ, Varuzza L, de B Pereira CA, Brentani H, Shmulevich I: **Simcluster: clustering enumeration gene expression data on the simplex space**. *BMC Bioinformatics* 2007, **8**:246.

3. Fawcett T: **An introduction to ROC analysis**. *Pattern Recogn. Lett.* 2006, **27**(8):861–874.
